# The Mission of the Profession of Medicine

**Published:** 1854-01

**Authors:** Benjamin R. Palmer

**Affiliations:** Professor of Descriptive and Surgical Anatomy


### MEDICINE AND SURGERY.

JANUARY, 1854. NEW SERIES. VOL. I.—NO. 1.

### ART. I.—THE MISSION OF THE PROFESSION OF MEDICINE.

*An Introductory Address, delivered before the Faculty and Students of the Medical Department of the University of Louisville, at the opening of the Session of* 1853–'54. *By* BENJAMIN R. PALMER, *M. D., Professor of Descriptive and Surgical Anatomy.*

A VERY general custom prevails among the American Schools of Medicine, by which, one lecture at least, somewhat formal and general in its character, is required as an introduction to the didactic courses which are to follow in the several departments of instruction. I confess that I can see no good reason for the establishment of this custom, neither can I appreciate the beneficial results which are supposed to require its continuance. Judging from my own observation, I am inclined to the opinion, that the occupancy of a professorial chair involves few duties more irksome to the incumbent than that which requires him, in his turn, to write and deliver "The Introductory." At least I am quite sure that the idea is always exceedingly disagreeable to me, and when, a few months since, the Dean of our Faculty advised me that I was expected to prepare for this occasion, I felt strongly inclined to ask for a release from so unpleasant a labor. But being persuaded that the respite would be but temporary, and that at length however ungraciously met, the duty must be encountered, I concluded to submit to the task imposed upon me, and appear before you this evening as the herald of the opening session of the University.

Then arose the somewhat anxious inquiry as to what should be the subject of the lecture. I knew that the whole field of topics suitable for such occasions had been thoroughly exhausted—that the ground had been cropped so often, that without a more fertilizing brain than mine, a very limited production must unavoidably be the result. It seems to me, that of all subjects which can be found to try the patience of a cultivated audience at this day, none will be as likely to prove threadbare, as a medical introduc. tory.

With these deprecatory remarks, allow me to ask your indulgence for a short time, while I offer a few reflections upon what I shall term the *Mission of the Profession of Medicine*, in other words, the results which legitimate medicine may be expected finally to accomplish, together with some considerations upon the mode in which they are to be attained.

Our profession has often been called a *conjectural art.* It has been frequently regarded as a collection of baseless hypothesis resulting from the unfounded speculations of misdirected genius, eliciting for a time the admiration of its disciples, and governing their conduct, to be succeeded in turn by new fancies equally chimerical, and equally transient.

That some foundation exists, or, I may better say, has existed for such calumny, cannot be denied. But the charge belongs more appropriately to the early days of medicine than to the present. Imperfections and errors have characterized all science and all art. They are the necessary attendants of progress. Even in the most exact of the sciences, the day has not yet arrived when no errors remain to be corrected, and no "false facts" to be exploded. It seems to me that there is no law of our nature more benign in its institution, than that which has created the necessity of laborious and untiring research in the great business of investigating the sublime principles which regulate the operations of matter and of mind, and their application in rendering them subservient to the highest interests of the race.

We are told in Holy Writ that "In the beginning God created the heaven and the earth, and the earth was without form and void, and darkness was upon the face of the deep." We know that certain great laws were instituted by the Creator for the government of this work of his hand, that these laws are fixed and unchanging—that after the lapse of ages they continue still their unvarying course. We know, too, that the crowning work of creation consisted in the production of man, with his elevation above all other beings of earth, with his intellectual and moral endowments constituting him, under God, their ruler and their master.

When the work of creation was finished, and man and brute, and tree and inanimate nature were left to fulfil the objects for which they were created, they were consigned by an infinite wisdom to the government of these immutable laws of their being.—There was no science then—no art—but the materials from which they were to be constructed existed then, as now, hidden, locked up, but not for ever, not beyond the grasp of that grandest and most mysterious of all the Creator's works—the human intellect. The sun shone then, as now. The silvery queen of night wheeled in her orbit, encircled by the glittering blazonry of heaven in the same unchanging round. The oak of the forest, springing from its germ, reared its massive branches toward the sun, and after defying the storms of centuries, withered, decayed, and was resolved into the elements from which it sprung. The rivers pursued their ceaseless courses to the sea. Tides ebbed and flowed as yesterday. Man and beast were as perfect in their structure as today. These organs so passing strange and wondrously beautiful in their complexity, were perfected then. The heart beats no truer now, the muscles obey the will no better now than they did in the forms of our first parents, when, sinless, they wandered amid the groves of Paradise, worshipping God in their unskilled but boundless admiration of his works, and wondering what manner of beings they were.

Philosophers have usually recognized a division of the laws of nature into moral and physical. The first has exclusive application to man as a moral and intelligent being. Under the second head may be grouped all that immense array of natural laws governing all created matter, beginning with man's animal nature and extending down to the minutest particle of the inorganic world. The knowledge of their nature, their relations, dependencies and operations constitutes human science. The direct application of this knowledge to the practical business of life becomes art. An acquaintance with the first, teaches man his own moral and intellectual nature, his relatious to his Maker and his fellow-men, his duties in this life and his existence in that which is to come.

All schemes of religion, all forms of government, all the social and domestic relations, whether in communities or in the narrower circle of family, are but the practical workings of the moral laws of our nature. When rightly understood and obeyed, virtue and happiness must be the necessary result; when perverted or neglected, vice and misery are the inevitable consequences. On the other hand, the physical laws, lying as they do at the foundation of all that is commonly termed natural science, which in turn constitutes the basis upon which all art is erected, are of an entirely different nature. While they have no immediate connection with the intellect, with the spiritual part of man, they are intimately concerned in the development, in the enlargement of the mind, and in the promotion of human happiness.

If we look at the early history of our race, commencing with that morning when man was ushered into being in the image of his God, it seems to me that there is no more striking fact to be observed than the great one connected with his early teaching.—Infinite wisdom saw that man required an instructor in those laws connected with his moral nature, that he could not by his own unaided powers determine his relations and responsibilities to his Maker, or solve the mysterious problem of futurity, and so we find from the dawn of creation down through the course of ages, the direct revelation of the will of Heaven, teaching us the great laws which govern our moral being, pointing out clearly our accountability to God and our duty to our fellow-men, urging us to good and warning us of evil, and finally assuring us with the comforting promise that our intellect, our soul is to live forever. Wise and good men assure us that this revelation contains all the teaching necessary to guide us to a life of virtue here, and one of happiness hereafter. We confidently accept this assurance, and devoutly rely upon this hope.

This teaching was explicit, for its importance and urgency admitted of no alternative. But no such instruction was vouchsafed in relation to the physical laws of nature; their discovery, their development and application to the purposes of life were left to the unaided intellect of man. It was evidently one great end designed by the Creator when he constituted his last work and gifted him with intelligence, that his marvelous powers of mind should be devoted to the immense task of discovering these hidden laws, that nature should be interrogated until she yielded her secrets as the reward of his toil; and for this wise and beneficent purpose man is bound to evince the deepest gratitude. It has not only served in the highest degree to develop the character, the energies and capacities of the race, but has been productive of immeasurable happiness.

It is far more interesting to the scholar to trace the slow but gradual progress of scientific discovery, to watch the almost imperceptible changes, at first so slight as scarcely to leave their impress, than to dwell upon those disjointed fragments of history which still exist as the evidences of human crimes and human passions. We are not surprised at the fact that during the early periods of the world, the progress of all scientific discovery was exceedingly slow. The reasons are too obvious to require notice. In the language of a distinguished member of our own profession, used in another connection: "It was light struggling with suffocating darkness, not for days, or weeks, or years, but for centuries upon centuries before the day-spring became manifest."

It seems to me not a chimerical or unreasonable opinion, that in the fullness of time all science and all art will become perfect, that the object of this world's creation will not be fulfilled, until not only the whole earth has become subject to the influences of civilization and religion, but all the laws of nature both moral and physical, in all their relations and dependencies shall have become clearly understood and applied to the great purpose of promoting the highest happiness of man. Is there not abundant reason for such an opinion? Does not the past history, and especially the present condition of the world warrant this expectation? How often are we startled, when some new secret of nature, hidden from the beginning, is stripped of the darkness which had enveloped it for ages, and takes its place among the acknowledged facts of science, exercising its expanding influence upon the practical business of life. Experience has already taught us not to wonder at discoveries which once, if predicted, would have excited the ridicule of all intelligent men. But a single century ago, men gazed at the lightning's flash and listened to the fearful crash of the thunderbolt in silent awe, regarding them only as magnificent but terrible illustrations of the power of God, and not unfrequently the sudden ministers of his wrath, little dreaming that the dread element would be deprived of its power for evil and rendered subservient to the highest interests of the race. The student of nature is constantly receiving the most gratifying reward for his toil and labor in exploring her secrets, and cannot fail to hope and believe that the long future will finish and perfect the work so auspiciously begun. When we read the pages of this world's history, which the geologist has opened for our admiration, during that long period of chaos and of night which lies behind the creation of man, and follow him step by step through the successive epochs during which the earth was preparing for its human inhabitants, and trace the unmistakeable evidence by which he demonstrates the almost countless ages which preceded the completion of the work, may we not anticipate that all the laws of nature and all dependent science will finally yield to human intellect and human labor?

I cannot resist the conviction, that inasmuch as long ages were required in the perfect preparation of the earth for its intelligent tenants, so in the long ages which are to follow the creation, the design of Omnipotence requires that all the laws pertaining to this work shall be known by his creatures, at least, so far as the knowledge shall tend to their advancement toward perfection and happiness.

In view of these considerations, which are all tending to the main topic of this lecture, the question arises, what may we hope, what may we expect will be finally accomplished by *scientific* medicine, and how is the work to be done? The aim and end of all legitimate labor in advancing our profession, must, of course, be the amelioration, the removal of human suffering resulting from disease or injury, and the prolongation of human existence.

Medicine is strictly a natural science, it is as strictly dependent upon, and governed by the fixed laws of nature as any other science. For its development and progress the discovery of these laws becomes as absolutely necessary. The investigation of their nature, relations, and application constitutes the field of labor which for centuries has been occupied by the true student of medicine, and which now lies open before him. All the laws of life, all the laws which govern living men and living animals, all the intricacies which constitute their mechanism, which produce and regulate their varied movements, giving to each organ its separate place and power, and yet combining them all in one harmonious whole, in fine, all the fixed and determined laws of structure and action, extending through all living beings, embracing every fact however minute and apparently trivial connected with the great problem of life, will, when fully understood, constitute the enduring basis of perfected medicine. The question now arises, can this finished, this perfected state, even of Anatomy and Physiology, be attained? Most certainly do we feel assured that anatomical science is now rapidly reaching its full and perfect stature. What is commonly termed descriptive human anatomy has received but few accessions to its facts for many years. We know but little more of bones and ligaments, muscles and tendons, blood vessels and nerves than our fathers knew. Their descriptions of size, form and relations will probably never be essentially improved. No one studies the attachments of a muscle, the ramifications of an artery, or the distribution of a nerve, with the expectation of becoming wiser than Meckel, or even Winslow was. Some curious and unusual arrangement, some strange and possibly unrecorded anomaly will be the only rewards of his labor. It is to the minuter fields of his science that the anatomist has for years devoted his original inquiries. That wonderful invention of man, that splendid exemplification of the subserviency of science to art, the microscope, enables the student of nature to extend his inquiries to the minuter particles of created matter. By it, he is becoming familiar with objects which once seemed entirely beyond the scope of human observation. It spreads before the eye of the Anatomist with startling distinctness the ultimate composition of every organized structure. Particles of matter whose diameters are measured by thousandths of an inch, are seen in their form, size and relations with a clearness not to be mistaken. They become established realities as much as the bones and muscles are, and take their place among the facts of science.

And here permit me to make a partial digression, or rather refer to a point which might have been omitted until a later period of the lecture.

There are some students of medicine, men whose minds are thoroughly imbued with the true spirit of progress, who are prone to attach no importance to knowledge which is not directly and immediately available in the exercise of their art. They are disposed to regard the labor expended in these minute investigations misapplied, thinking that but little importance can ever be attached to their results. Whether the human blood disc has a diameter of the three thousandth, or ten thousandth of an inch, or whether it has any determinate diameter is in their view a matter of no consequence.

When the Microscopist informs them that the ultimate muscular fibrilla has a breadth of the nine-thousandth of an inch, they are disposed to regard the fact too trivial for the attention of a grave student of medicine. But even if we cannot now see any direct practical importance coming from this knowledge, are we justified in rejecting it as useless, in regarding it as merely adapted for the exercise of the curiosity of the idle, and not as the legitimate domain of the true votary of medicine? It requires the review of but a few years in the history of medical progress, to establish beyond all cavil the value of such studies. When the animal chemist was teaching our predecessors the composition of the fluids, the practical men of that day regarded his instructions as rather a matter of scientific curioisty and amusement, than as directly connected with their duties in ministering to sick and dying men. They did not see, as they now do, the importance of knowing, that healthy blood contains three parts in the thousand, of the element called fibrin. When the microscopist first called attention to the forms and dimensions of certain crystaline products, occasionally found in another fluid, they regarded them rather as beautiful illustrations of some of the wonderful works which nature performs in her secret laboratories, than as directly connected with the knowledge and treatment of disease.

Innumerable illustrations might be adduced to show how much practical medicine has *already* been indebted to these researches of the minute anatomist; and although our clouded vision may not yet be able to distinguish an importance or see a relation between many of these facts, and the greater and more obvious phenomena with which we are familiar, still let us not reject them as useless; let us not abandon their further investigation as unprofitable. I can not believe that there is any knowledge, however minute connected with the living mechanism, which will not eventually prove to have a direct relation to the practical duties of our profession.

It seems to me that all facts in all science are to be sought and treasured, not merely as curiosities for the amusement of the scholar's hours of leisure, but as having an ultimate bearing in the advancement of our race toward perfection and happiness.

In these minute studies, serious difficulties must be encountered and mistaken opinions must, necessarily, occasionally result from labor carelessly or imperfectly performed. In a comparatively new field, errors are almost unavoidable, but the same spirit of progress which quickens the zeal of the faithful student, and occasionally leads to hasty and erroneous conclusions, will in the end correct and establish them in truth.

It cannot be accounted as visionary, when we express the confident opinion, that at some period (we know not how far distant,) the labors of the microscopist, and the researches of the chemist will complete and perfect this department of our science; when man will know his own physical structure in all its wonderful beauty and complexity. There is no insuperable difficulty here; from the point where we now stand, we can see our way not dimly but clearly to the end.

And now may we expect thus ever, thus completely to comprehend the mysterious workings of this mechanism. Can its physiology, its life be fathomed in all its heights and depths? Can we ever hope to understand all the great laws of its being; those which govern its incipiency, its growth, its maturity, its decay, and its death? These are mightier subjects far than any which engage the anatomist's attention in his labors. They require for their development more untiring labor, more unbounded devotion.

The Physiologist must explore the whole realm of living matter; he cannot otherwise determine the varied laws of his own structure, for he finds analogies and relations distinctly running through the whole range of organized existence. Many of the operations of life and the laws which govern them are the same, whether observed in man, or brute, or tree, or the humble plant which marks the confines of the living world. With this extensive field of research, presenting as it does an almost endless multitude of facts, all important as illustrating his science, may the Physiologist ever hope to complete his work, may he ever hope to discover and establish all the phenomena, all the laws of life, to teach men how they live, and how they die. Life and death are among the most mysterious of the results of creation. The essence of life, as it is sometimes termed, may, and probably will, be always beyond the reach of finite understanding. Life, intellect, soul—these are terms by which we embody our idea of things immaterial, which we only know by their workings, by their results. And yet in this respect they are not alone. We speak of gravity, electricity and magnetism, as something real, something absolute, but the wisest philosopher only points to their results; he knows nothing else of their nature, of their essence. It does not seem to me at all necessary for our purpose that we should be able to comprehend this mysterious essence; it is enough for us to know all its laws and all their phenomena.

We need not fathom the essentiality of mind, but we should know how it uses its material instruments, how it controls their workings, and how they in turn modify and influence it. We shall probably never know why a nerve thread, and that alone, can convey a volition or a sensation, or why a muscular fibre alone is able to contract in obedience to the will. These are facts doubtless beyond our reach; and we cannot see that their knowledge would be important; but we must learn how these organs work, the laws to which they are obedient and the results of their actions.

The object of the Physiologist is not to comprehend what life is, but its laws; or in other words, when we shall have learned the uses of all parts of the living structure, even their minutest atoms, when we shall have found ont their whole history from the beginning to the end, when we shall have ascertained all the circumstances which influence their actions, and what these actions are, then may the laws of life be regarded as discovered, their phenomena understood, and the science of physiology perfected. And can we doubt that such will be the ultimate reward of scientific labor? Does not the past, and especially the history of the last few years of our professional progress point to this conclusion? If the time admitted, abundant illustrations might be given showing how far we have already succeeded in unlocking what were once regarded as the impenetrable secrets of nature, in lifting the veil which shrouded her mysteries and revealing the wonderful phenomena of life. Although very much remains to be done before this great work shall be accomplished, yet the conclusion is irresistible, that in due time the science of Physiology like that of Anatomy will be complete, that the actions as well as the structure of living beings will be clearly understood.

I have considered somewhat fully these two elementary sciences, and for obvious reasons. The scientific physician always recognizes their fundamental importance, he sees that no true progress in the practical branches of medicine can be made in advance of these, that no reliable and enduring structure can be reared upon an insecure foundation. Doubtless important knowledge of disease and remedies has been attained to, in ignorance of the physiology of the part diseased, but no hope can ever be entertained of perfecting this knowledge, while the least obscurity pertains to any part of its laws of healthy structure and action. A scheme of practical medicine erected upon any other foundation, is in worse condition than the house discribed in Holy Scripture, which was built upon sand, for *that* stood, until storms came, and winds blew while the medical fabric, as has been often and abundantly proved falls of its own weight; it needs no storms for its destruction, for however slight and airy the structure, its insecurity and want of support require no causes from without to produce its ruin.

And now we approach what is perhaps the most difficult part of our subject. What are we to expect, what may we hope, will be the result of all the toil and labor which now so eminently characterize the *true* disciples of practical medicine? what may we hope will be known, in the progress of time, of disease and its cure? I shall here use the term Practical Medicine with more latitude than is usually assigned to it. I shall consider it as embracing not only diseases and their treatment, but surgery, medicines, obstetrics, in fine all the practical branches of our science.

The question arises, what are we to regard as perfection in practical medicine? What progress must be made before this condition can be attained? Doubtless the wild dreams of the Alchemists have passed away forever. The visionary enthusiasts of the middle ages who spent long years in idle and fruitless labor for the discovery of the *elixir vitæ*, a something capable of reproducing and continuing a period of perpetual youth, were never enrolled among the true disciples of medicine. They were the empirics of their day, and like others of their class, have passed away and left few traces of their existence save the record of their folly.

While this earth is inhabited by men, they must obey that great law of their being which "fortells the ending of mortality." Unchanging and unchangeable is the memorable denunciation, "dust thou art, and unto dust shalt thou return." The perfection of practical medicine does not necessarily imply that all diseases should be deprived of their fatality, and human life in all cases protracted to its extremest limit. It only requires that *all* the laws of *all* diseases should be fully known, and that the nature and action of all remedial agents should be fully understood. It cannot be doubted, that natural science comprises within its broad domain all the facts pertaining to the diseased as well as to the healthy organs, that *their* changed structure and perverted actions obey laws just as fixed and unerring as those simpler and more easily understood, which make our physiology. There can be no doubt that with all the circumstances alike, antecedent and surrounding, the same disease would always present the same peculiarities. In other words, we believe that every disease has its fixed laws, that the varieties which are observed, are dependent upon conditions either pertaining to the patient himself or to the cause which produced it, or those which may modify its course and action, and it may be fairly inferred that these causes, these circumstances, are uniform in their result.

And again, we inquire, can all the laws of disease be compassed? Are all the facts constituting this immense field of science within reach of the human intellect? It seems to me immeasurably the grandest and broadest work which God has assigned to the children of men. It has a higher, a nobler aim and end than any other human science, and the herculean task of investigating and determining its facts is proportionate to the immensity of the result.

Let us take a brief view of the work to be done, and then we can better comprehend the amount of labor before us, and better judge of the probable issue. In investigating any disease, we find the range of inquiry exceedingly broad. A great multitude of facts must be observed with most scrupulous care; the observations must be repeated again and again, that all doubt of their accuracy, be removed. These facts must be arranged, compared and analyzed, so that from them, at length the laws of the disease may be deduced. They must also be repeated under every variety of circumstances, which can exert any influence upon the results; in the different sexes, different seasons of the year, and in differ-regions of the earth. Regard, too, must be had to the different races of men, and to peculiarities in the same race. So too the age, occupation, and habits of life, all require the student's investigation. These, and other circumstances which might be adduced would convey some idea of the conditions which bind all investigations which have for their object the determination of the laws of disease.

Prominent among these laws, prominent for their vast importance, as well as for the difficulties which in many cases surround and obstruct our passage, are the causes of disease. We already possess a large amount of knowledge in this important division of our science. In not a few diseases, perhaps all that is valuable, all that ever will be known, is fully understood; while in others, a partial but still useful degree of knowledge requires a vast amount of labor for its full development. In certain diseases of a purely contagious character, our acquaintance with the cause is definite. We know that during their progress a poison is generated which reproduces a similar disease in persons subjected to its influence. This poison will produce no other form of disease, nor can the malady be produced by any other cause. Small-pox is never known to originate except from exposure to its peculiar virus. On the other hand, certain diseases termed epidemic, are dependent upon causes but imperfectly understood.

We do not yet fully know, why Asiatic cholera should expend its desolating influence upon one neighborhood, sweeping from the earth in a few short weeks, its thousands of victims, while in the immediate vicinity, the destroyer passes by, leaving cities and villages comparatively unharmed. Yet even here, some useful knowledge has been obtained, a clew has been reached, we would fain believe, by which the mystery will at length be unravelled. During the early history of your own beautiful valley, when the foot of the white man first pressed the banks of the "lonely Ohio," when the axe of the settler and the rifle of the hunter gave evidence of the progress of our untiring, unresistable race, in its resistless march across a hemisphere, the pioneer found amid the luxuriant forests of these fertile plains, a foe more deadly than the red man, more difficult to be encountered than the wildest beast of the unbroken wilderness. Pestilence walked in darkness and wasted at the noon-day. Nearly an entire generation bowed to its influence and was swept away. With the advance of civilization, with the peopling of the earth's surface and its consequent cultivation, the pestilence has slowly, but steadily receded, and now the stalwart forms of hardy men, and the glowing cheeks of beauteous women, tell full surely that the genius of health inhabits your borders. And what produced this fearful desolation? Why did the early settlers of almost all parts of our broad land become its victims? Physicians term the cause *malaria*, and why? The breeze of the morning was as fragrant and balmy, the zephyr of evening was as cool and refreshing to the brow of the weary laborer as now. The chemist finds no difference in the composition of the atmosphere, whether he studies it amid the eternal glaciers of the frozen Alps, or beneath the burning sun of the tropics, or amid those pestilential marshes whose noxious exhalations almost depopulate the fairest provinces of the earth.

We have thus far interrogated him in vain for a solution of the enigma. Our knowledge extends only to some of the conditions which surround this dread cause of mortality. We feel that we know whence it originates and how it is removed. That it is a something, too subtle for the chemist's present means of reach, emanating from the earth and mingling with the air we breathe, we are persuaded; but what it is, future discoverers must teach.

I have thus briefly referred to what may be considered the two extremes of the known and the unknown causes of disease. Between these, the subject might be extended almost illimitably, but it is unnecessary. Next to disease itself, its nature, its progress, its termination, in all its various forms, what an immense amount of knowledge is already ours, and what a vast field of study is yet before us. There is no malady incident to civilized man with which we are not to a considerable extent familiar, and there is probably none which does not require more labor for its perfect comprehension. We must know every change produced from the beginning of every disease to its end. Familiarity with the slightest alteration in the composition of every fluid, in the form, size and structure of every atom of the solid constituents is requisite, before our knowledge of the nature of any disease can be considered perfect, and this too under every variety of circumstances which ever occur to influence its course and action.

With this knowledge, coupled with an acquaintance with the outward symptoms, the physician will read with his patient before him, as reflected in a mirror, all the silent workings within; he will be able day by day to anticipate the changes of the morrow, and apply with a purpose *never* conjectural, all the resources of his art. With a full knowledge of all the laws of all diseases, he will of course be able to determine whether any given malady is curable, or whether from its nature it must defy all skill. We acknowledge with melancholy sadness, that amid the long catalogue of diseases, some yet exist, which baffle all our efforts for their cure. Again and again we struggle manfully but fruitlessly for the mastery, and when now and then a solitary victim is rescued from the destroying angel's grasp, we almost wonder at the unusual occurrence. How hopelessly does the physician stand and gaze upon the poor wretch writhing under the agonizing tortures of hydrophobia, and how powerless does he feel; how little comfort can he give to the victim of cancer. He knows that silently, perhaps slowly, but almost surely it is sapping the foundations of life, he knows that the living current is poisoned, and is polluting and destroying the whole fabric. He reads its progress in the impaired strength, the wasted form, the ghastly face and sunken eye, and can he minister no aid? The surgeon proposes its removal, it is his only hope. It probably will prolong existence, and for a time assuage its torments; it may cure, but of that he can speak but doubtingly, and still it is the only hope. Thus it is now, but when knowledge here shall be complete, it may prove that its law is death, or it may chance, that when all its causes are crearly understood, when science shall have fathomed the slightest changes which mark its beginning, that art may be able to furnish its preventive or its cure, and another triumph be added to the long list, which now adorns the pages of our profession.

When we inquire into the probabilities of this final perfection in the knowledge of disease, when we ask whether this vast result can ever be attained, it is fitting and right that we examine the past and the present condition of our science; for of the future we may judge of the past. Have we already made such advances as to justify these expectations? A single illustration must suffice for this purpose, and I shall take a disease of great frequency and great importance, inflammation of the lungs, popularly termed lung fever, a disease which has long been recognized and named. Half a century ago, and physicians knew merely that a process, termed inflammation, was liable to attack these important organs. They could sometimes, but by no means uniformly, distinguish it from diseases of neighboring parts. They had a general but vague idea of its progress. They dreaded its tendencies to fatality, and when such an event occurred, they supposed that some kind of disorganization had been the cause. And how is it now? The skilled physician recognizes the presence of the disease in its very beginning. He always distinguishes it clearly from all other affections, and ascertains at the onset the exact point of the organ invaded. However small the inflamed part may be, it does not escape his notice. Day by day he watches its progress, and if it advance, he can mark the dividing line between the diseased and healthy parts. As it changes its character he marks it with unerring certainty, and can even anticipate the change. He knows, too, the workings of the disease within; the alteration in the composition of the blood; the different manner in which it pursues its busy round. He knows how the solid structures are changed, even to their microscopic atoms. He sees, as in a glass, the blood vessels pouring out a portion of their contents, he sees these mingled with, and blocking up the delicate tissues called air cells, thus hurrying the breathing, and rendering it imperfect; he can comprehend the purple hue of the face, the delirious wandering of the intellect, warning him of danger, not because the brain is involved in the disease, but because the laboring lungs are no longer able to fit the blood for its circulation through the organ of the mind. In short it would seem as though there could be comparatively but little more in the nature of this disease for the labor of future discoverers. Nor does it stand in this respect alone; a long list could be added, in which the progress of investigation has carried us far, very far, as we believe, toward the end, although in no disease, as it seems to me, can we regard our knowledge yet as complete.

The last half century has been eminently characterized by the rapid progress of all science and all art. The world is familiar with the wondrous changes which have resulted from this progress. Almost all men recognize and appreciate the immeasurable amount of comfort and happiness for which they are indebted to the unceasing toil of the devoted student of nature. We believe we know that no science has been characterized by more untiring labor than ours, we are confident that in none has greater progress been made. The question will naturally be asked, has this progress in the knowledge of the laws of life, of health, of disease and death, been attended with a corresponding advance in the great and ultimate object towards which all our labors tend?—Has this progress enlarged our means for the prevention and cure of disease? Has it enabled us more successfully to guard against its approach, to resist its attacks, and avert its fatal termination? Most abundant and satisfactory answers can be given to these questions. Examine the statictics of hospitals, and they will show you a steady and marked decrease in their tables of mortality.—Look at the statistics of scientific medicine every where, and you will find them filled with the triumphs of our art. If the time permitted and the occasion required it, we could point you to many diseases once regarded as nearly hopelessly fatal, which now yield readily to our skill. The Surgeon could tell you of limbs now saved, where but a few years ago their removal was considered unavoidable. He could show you the subjects of deformities which once followed their victims from the cradle to the grave, now restored by his skill to the perfect form of perfect men. Sight to the blind, strength to the lame and impotent, life to the once almost hopelessly diseased, are the rewards of his labors.—Equally important practical results have attended the researches in another branch of our profession. All reports, all statistical records show conclusively, that great advances have been made during the last half century, in the prevention of disease, in the relief of its pangs and in the prolongation of life; and for these inestimable results the world is indebted to our science.

In view of what has been done, and especially in view of the wonderful advance of medicine during the last fifty years, and of the untiring zeal of the host of true disciples who now lead the van of progress and discovery, it seems to me not unreasonable to believe, that the day will come, when all the laws of disease will be known, it seems to me that the past and the present both point clearly to this result, that in our science, it is the design or Providence that all the laws upon which it is based shall eventually be known and understood in all their relations, at least so far as their knowledge will conduce to human happiness.

The road to this great end has already been sufficiently indicated. Nature must be observed and interrogated until her hidden secrets are brought to light. Our studies are conducted in the same manner as in the other natural sciences. There is no mystery in our labors; careful observation and cautious induction comprise it all.

The tendency to speculation is rapidly vanishing, and the danger of wandering from the right path has nearly passed away.—All that is valuable in our knowledge, all that has stood the test of time, or will endure the ordeal of future investigations, is the outgrowth of the labors of the disciples of legitimate medicine.—It is only from the *regular* priesthood who minister at her altars, that our science derives lessons of wisdom and truth. All the fanciful speculations, all the wild schemes for the cure of disease which have emanated from the brains of foolish empirics have proved as baseless and as transient as the visions of the night.—One follows another, and for a time amuses the credulous and then passes away never to be revived. So hath it always been, so must it always be, from the very nature of things it can never be otherwise. The empiric knows naught of nature's laws and cares naught, he never dreamed of the scholar's love of knowledge for the sake of knowledge. The "*sacra fames auri*," is the only law, the only principle which incites him to labor. But as his life is without profit to science, it is pleasing to feel that it does no evil. It can create but a passing eddy, a ripple upon the surface which is soon swept away and lost forever, in the rapid progress of the broad and deep current of scientific medicine.

